# Low Birth Weight Is Associated with a Decreased Overall Adult Health Status and Reproductive Capability – Results of a Cross-Sectional Study in Primary Infertile Patients

**DOI:** 10.1371/journal.pone.0166728

**Published:** 2016-11-28

**Authors:** Luca Boeri, Eugenio Ventimiglia, Paolo Capogrosso, Silvia Ippolito, Angela Pecoraro, Marco Paciotti, Roberta Scano, Alessandro Galdini, Luca Valsecchi, Enrico Papaleo, Francesco Montorsi, Andrea Salonia

**Affiliations:** 1 Division of Experimental Oncology/Unit of Urology; URI; IRCCS Ospedale San Raffaele, Milan, Italy; 2 Vita-Salute San Raffaele University, Milan, Italy; 3 Department of Obstetrics and Gynaecology, IRCCS Ospedale San Raffaele, Milan, Italy; 4 Infertility Unit, Unit of Obstetrics/Gynecology, IRCCS Ospedale San Raffaele, Milan, Italy; University of California San Francisco, UNITED STATES

## Abstract

Individuals born with low birth weight (LBW) risk cardiometabolic complications later in life. However the impact of LBW on general health status and male reproductive function has been scantly analysed. We investigated the clinical and seminal impact of different birth weights (BW) in white-European men presenting for primary couple’s infertility. Demographic, clinical, and laboratory data from 827 primary infertile men were compared with those of 373 consecutive fertile men. Patients with BW ≤2500, 2500–4200, and ≥4200gr were classified as having LBW, normal (NBW), and high BW (HBW), respectively. Health-significant comorbidities were scored with the Charlson Comorbidity Index (CCI). Testicular volume was assessed with a Prader orchidometer. Semen analysis values were assessed based on 2010 WHO reference criteria. Descriptive statistics and regression models tested associations between semen parameters, clinical characteristics and BW categories. LBW, NBW and HBW were found in 71 (8.6%), 651 (78.7%) and 105 (12.7%) infertile men, respectively. LBW was more frequent in infertile patients than fertile men (p = 0.002). Infertile patients with LBW had a higher rate of comorbidities (p = 0.003), lower mean testicular volume (p = 0.007), higher FSH (p = 0.02) and lower tT levels (p = 0.04) compared to other BW groups. Higher rates of asthenozoospermia (p = 0.02) and teratozoospermia (p = 0.03) were also found in LBW men. At logistic regression models, LBW was univariably associated with pathologic progressive motility (p≤0.02) and pathologic sperm morphology (p<0.005). At multivariable logistic regression analysis, LBW achieved independent predictor status for both lower sperm motility and pathologic sperm morphology (all p≤0.04). Only LBW independently predicted higher CCI values (p<0.001). In conclusion, we found that LBW was more frequent in infertile than in fertile men. Infertile individuals with LBW showed a higher rate of comorbidities and significantly worse clinical, endocrine and semen parameters compared to other BW groups.

## Introduction

The recent discovery that people with chronic disease develop differently from others during fetal life and childhood has led to a new “developmental” model for a group of diseases, thus including coronary heart disease, stroke, high blood pressure and type 2 diabetes mellitus [1]. The Developmental Origins of Health and Disease (DOHAD) address the concept of developmental plasticity, a critical time during development, typically occurring in utero, when a system is plastic and sensitive to various stimuli such as the nutritional, hormonal and metabolic environment. This plasticity may help explain the range of different physiological or morphological states that arise in response to different developmental conditions [2]. The fact that pre-natal factors play a role in gonadal development [3,4] and the replication of Sertoli cells in prenatal and postnatal life has already been established [3]. However, the underlying mechanisms regulating the relationship between the early developmental environment and postnatal reproductive development and function are unclear [5]. Given the difficulties to retrospectively assess in utero nutrition in humans, birth weight (BW) is commonly used as a proxy for nutritional conditions during fetal life. Indeed, although data are not univocal epidemiological studies have shown that individuals born small for their gestational age (SGA) are at an increased risk of developing insulin resistance, cardiovascular disease and metabolic syndrome as adults [6]. Other endocrine pathways, such as the hypothalamic-pituitary-gonadal axis, have also been implicated; results of the association between birth weight and gonadal function in both men and women are still conflicting. In this context, Francois *et al*. reported that men born with SGA are more likely to present subfertility [7]. Furthermore, a number of authors have shown reduced gonadal function in males born with SGA, as compared with males born of appropriate size for their gestational age [8–10]. Recently, Faure *et al*. found that BW was associated with sperm DNA fragmentation and inversely correlated with total sperm count [11]. Conversely, other authors have failed to find significant associations between BW and gonadal function [12–15], even with the observation that there is no correlation between BW and male infertility [16,17]. Likewise, not unequivocal studies in women demonstrated impaired gonadal function and increased prevalence of anovulation in adolescent girls born with SGA [18–21].

These conflicting observations, along with the lack of previous clinical evidence considering the potential impact of LBW on general health status and overall male reproductive function prompted us to investigate the eventual role of different categories of BW in male infertility, specifically assessing (i) prevalence of LBW, (ii) its correlations with clinical characteristics, and (iii) its impact on semen and hormonal parameters in a homogeneous cohort of white-European men presenting for primary couple’s infertility. Moreover, clinical and BW of primary infertile men were compared with those of a homogeneous cohort of age-comparable fertile men.

## Materials and Methods

### Patients

The analyses of this cross-sectional study were based on a sample of 850 consecutive white-European men assessed at a single academic centre for primary couple’s infertility (non-interracial infertile couples only) between January 2012 and September 2014. According to the World Health Organisation (WHO) criteria, infertility was defined as not conceiving a pregnancy after at least 12 months of unprotected intercourse regardless of whether or not a pregnancy ultimately occurred [22]. Primary infertility is defined when a couple has never been able to conceive [22]. Infertile patients were enrolled if they were between 18 and 60 years of age and had only male factor infertility (MFI); MFI was defined after a comprehensive gynecological evaluation of the female partners. Patients underwent at least two consecutive semen analyses, both showing below standard values for normal semen parameters according to the WHO criteria [23].

Patients were assessed with a thorough medical history including age and comorbidities. Comorbidities were scored with the Charlson Comorbidity Index (CCI) [24]. We used the International Classification of Diseases, 9th revision. For the specific purpose of the analysis, CCI was categorised as 0 or ≥1. All individuals were sexually active, with at least four sexual intercourses per month. Weight and height were measured for each participant, and body mass index (BMI), defined as weight in kilograms by height in square meters, was calculated. Body mass index was considered for each patient using the cut offs proposed by the National Institutes of Health (NIH): normal weight (18.5–24.9), overweight (25.0–29.9), and class ≥1 obesity (≥30.0). Lifestyle factors potentially related with any impairment of semen quality were carefully assessed. Smoking status was divided into two categories, thus including current/ex smokers vs. never smokers. Alcohol consumption was stratified using the cut-off values based on the definition by National Institute on Alcohol Abuse and Alcoholism as abstainers (non alcohol consumption history), moderate drinkers (up to 2 drinks/day), and heavy drinkers (>2 drinks/day) [25]. Colour-Doppler ultrasound was used to detect spermatic vein reflux and to classify the grade of varicocele in infertile patients. All individuals were born at term (37–41 weeks of amenorrhea). None of the participants reported having undescended testes at birth. Birth weight was collected from the childhood health records of each individual. Patients with BW ≤ 2500, 2500–4200, and ≥ 4200 gr were classified as having low birth weight (LBW), normal birth weight (NBW) and high birth weight (HBW), respectively [26]. Testes volume was assessed through a Prader orchidometer. Infertile patients underwent at least two consecutive semen analyses. Semen samples were collected by masturbation and analysed within 2 h according to the WHO criteria [23]. As a main entry criterion for the study, only patients with a complete data collection were included; therefore, patients with incomplete medical history or without a detailed self-reported birth weight (n = 23 [3%]) were excluded. A total of 827 patients (97.3%) were included in the final analyses.

We also collected clinical data from 373 fertile white-European men who had fathered at least one child, spontaneously conceived, with a time to pregnancy within 12 months. These men were recruited via their partners who had been new and expectant mothers in our department of Obstetrics and Gynaecology. For the specific purpose of the study we collected only clinical data from the fertile group of individuals (e.g. age, BMI, CCI, birth weight).

Venous blood samples were drawn from each infertile patient between 7 am and 11 am after an overnight fast. In all cases, fasting glucose levels were measured via a glucose oxidase method (Aeroset Abbott, Rome, Italy). Total cholesterol, HDL-C, and triglyceride levels were measured with the automated enzymatic colorimetric method (Aeroset Abbott, Rome, Italy). Impaired HDL-C values were considered if HDL-C < 40 mg/dl. Hypercholesterolemia (total cholesterol level ≥200 mg/dl) and hypertriglyceridemia (triglycerides ≥150 mg/dl) were assessed [27]. Follicle-stimulating hormone (FSH); luteinising hormone (LH), prolactin (PRL), thyroid-stimulating hormone (TSH), and 17-β-estradiol (E_2_) were measured using a heterogeneous competitive magnetic separation assay (Bayer Immuno 1 System, Bayer Corp., Tarrytown, NY, USA). Inhibin B (InhB) and anti-Müllerian hormone (AMH) were measured with an enzyme-linked immunosorbent assay (Beckman Coulter AMH Gen II ELISA). Total testosterone (tT) levels were measured via a direct chemiluminescence immunoassay (ADVIA Centaur; Siemens Medical Solutions Diagnostics, Deerfield, IL, USA), and sex hormone-binding globulin (SHBG) levels were measured via a solid-phase chemiluminescent immunometric assay on Immulite 2000 (Medical Systems SpA, Genoa, Italy). Hypogonadism was defined as tT less than 3 ng/ml [28]. The same laboratory was used for the analysis of all parameters for all patients.

### Ethical approval

Data collection was carried out following the principles outlined in the Declaration of Helsinki; after approval of the IRCCS San Raffaele Hospital’s Ethical Committee, all patients signed an informed consent agreeing to supply their own anonymous data for this and future studies.

### Statistical analyses

Data are presented as means (SD; ranges). The statistical significance of differences in means and proportions was tested with the one-way analysis of variance (ANOVA) and Pearson chi-square test, respectively. A 95% confidence interval was estimated for the association of categorical parameters. Exploratory analyses were initially applied to all variables; variables were retained for analysis when deemed clinically significant to the results. Logistic regression univariable analysis (UVA) and multivariable analysis (MVA) tested the associations between clinical and laboratory predictors (e.g. age, BW, FSH, CCI, varicocele, BMI, cigarette smoking and alcohol intake) and pathologic semen parameters as defined by the WHO 2010 criteria [23]. Further logistic regression models tested the association between predictors and the presence of comorbidities (CCI≥1). Statistical tests were performed using SPSS v.19 (IBM Corp., Armonk, NY, USA). All tests were two sided, with a significance level set at 0.05.

## Results

Table 1 lists the characteristics and the descriptive statistics of the entire cohort of individuals. Fertile and infertile men did not differ in terms of age. Conversely, infertile men showed a higher mean BMI value (p = 0.02) and a trend toward a greater proportion of BMI values suggestive of NIH class ≥1 obesity (p = 0.048) compared to fertile men. There were no differences in terms of lifestyle factors (smoke and alcohol consumption) between groups. Moreover, the presence of clinically significant comorbidities (CCI≥1) was more frequently reported in infertile patients, rather than fertile men (p = 0.039).

**Table 1:** Characteristics and descriptive statistics of all participants (No. = 1200).

	Infertile	Fertile	p value (F)*
No. of individuals	827	373	
Age (years)			0.32 (0.95)
Mean (SD)	36.5 (5.3)	36.2 (5.4)	
Range	18–60	22–55	
Categorized age [No. (%)]			0.30 (χ^2^, 3.62)
18–25	14 (1.7)	6 (1.6)	
26–35	384 (46.4)	170 (45.6)	
36–49	400 (48.4)	191 (51.2)	
≥50	29 (3.5)	6 (1.6)	
Birth Weight (gr)			0.038 (4.32)
Mean (SD)	3447.5 (649.4)	3529.7 (597.1)	
Range	1100–5500	650–5300	
Birth Weight [No. (%)]			0.002 (χ^2^, 12.31)
LBW	71 (8.6)	12 (3.2)	
NBW	651 (78.7)	318 (85.3)	
HBW	105 (12.7)	43 (11.5)	
BMI (kg/m^2^)			0.02 (5.39)
Mean (SD)	25.8 (3.7)	25.2 (3.6)	
Range	18.1–44.98	16.6–50.6	
Categorized BMI [No. (%)]			0.048 (χ^2^, 6.08)
18.5–24.9	390 (47.2)	187 (50.1)	
25–29.9	341 (41.2)	160 (42.9)	
≥30	96 (11.6)	28 (7.0)	
CCI [No. (%)]			0.039 (χ^2^, 4.38)
CCI 0	751 (90.8)	352 (94.4)	
CCI≥ 1	76 (9.2)	21 (5.6)	
Alcohol [No. (%)]			0.91 (χ^2^, 0.05)
Abstainers	182 (22.0)	80 (21.4)	
Moderate drinkers	487 (58.9)	221 (59.2)	
Heavy drinkers	158 (19.1)	72 (19.3)	
Cigarette smoking [No. (%)]			0.62 (χ^2^, 0.46)
Current/ex smokers	263 (31.8)	110 (29.5)	
Never smokers	564 (68.2)	263 (70.5)	
Testis volume (Prader estimation)			
Right testis			
Mean (SD)	16.7 (5.9)		
Range	2–25		
Left testis			
Mean (SD)	15.99 (6.0)		
Range	2–25		
Varicocele [No. (%)]	437 (52.8)		
Hypercholesterolemia [No. (%)]	145 (17.5)		
Hypertrigliceridemia [No. (%)]	35 (4.2)		
Impaired HDL-C [No. (%)]	111 (13.4)		
FSH (mUI/mL)			
Mean (SD)	9.2 (12.1)		
Range	0.1–198.4		
LH (mUI/mL)			
Mean (SD)	5.5 (7.6)		
Range	0.1–433.9		
InhB (pg/mL)			
Mean (SD)	113.3 (82.3)		
Range	0.1–439.4		
AMH (ng/mL)			
Mean (SD)	6.3 (7.0)		
Range	0.1–96.3		
tT (ng/mL)			
Mean (SD)	4.8 (1.8)		
Range	0.0–16.8		
tT <3 ng/mL [No. (%)]	99 (11.9)		
E_2_ (pg/mL)			
Mean (SD)	32.9 (14.2)		
Range	5.0–129.0		
SHBG (nmol/L)			
Mean (SD)	33.2 (13.3)		
Range	6.0–95.0		
PRL (ng/mL)			
Mean (SD)	9.6 (7.3)		
Range	1.0–135.5		
TSH (μUI/mL)			
Mean (SD)	2.1 (3.6)		
Range	0.0–77.9		
Semen volume (mL)			
Mean (SD)	3.0 (1.7)		
Range	0.2–9.5		
Semen volume <1.5 mL [No. (%)]	165 (20.1)		
Sperm concentration			
Mean (SD)	33.4 (40.5)		
Range	0.5–266.7		
Sperm concentration ≤15x10^6^/mL [No. (%)]	339 (41.0)		
Progressive motility			
Mean (SD)	26.1 (20.1)		
Range	0.0–94.0		
Progressive motility ≤32% [No. (%)]	434 (52.4)		
Normal morphology			
Mean (SD)	5.7 (11.3)		
Range	0.0–92.0		
Normal morphology ≤4% [No. (%)]	495 (59.9)		

BMI = body mass index; CCI = Charlson Comorbidity Index; LBW = low birth weight; NBW = normal birth weight; HBW = high birth weight

*P value according to chi-square test or analysis of variance (ANOVA), as indicated

As a whole, LBW was found in 8.6% of patients and 3.2% of fertile individuals (Table 1); mean BW was significantly lower in infertile patients (p = 0.038) and LBW was significantly more frequent in the infertile group of men (p = 0.002).

Table 2 depicts the characteristics and the descriptive statistics of infertile patients according to the established BW categories. Patients did not differ in terms of age among groups. Overall, BMI values were higher in HBW men (p = 0.03), who also showed a greater proportion of BMI value suggestive for NIH class ≥1 obesity (p = 0.02).

**Table 2:** Sociodemographic characteristics and descriptive statistics of infertile patients according to birth weight.

	LBW	NBW	HBW	p value (F)*
No. of patients [No. (%)]	71 (8.6)	651 (78.7)	105 (12.7)	
Age (years)				0.21 (1.61)
Mean (SD)	36.3 (6.1)	36.4 (5.6)	37.5 (6.8)	
Range	18–53	18–60	25–60	
Categorized age [No. (%)]				0.06 (χ^2^, 12.15)
18–25	4 (0.5)	9 (1.1)	1 (0.1)	
26–35	32 (3.9)	309 (37.4)	43 (5.2)	
36–49	32 (3.9)	314 (38.0)	54 (6.5)	
≥50	3 (0.4)	19 (2.3)	7 (0.8)	
BMI (kg/m^2^)				0.03 (3.48)
Mean (SD)	25.6 (3.9)	25.7 (3.6)	26.7 (4.0)	
Range	19.3–35.0	18.1–45.0	18.6–40.8	
Categorized BMI [No. (%)]				0.02 (χ^2^, 11.6)
18.5–24.9	33 (46.5)	321 (49.3)	36 (34.3)	
25–29.9	27 (38.0)	264 (40.6)	50 (47.6)	
≥30	11 (15.5)	66 (10.1)	19 (18.1)	
CCI [No. (%)]				0.03 (χ^2^, 6.82)
CCI 0	59 (83.1)	599 (92.0)	93 (88.6)	
CCI≥ 1	12 (16.9)	52 (8.0)	12 (11.4)	
Testis volume (Prader estimation)				
Right testis				0.08 (2.56)
Mean (SD)	15.8 (7.12)	16.6 (5.8)	17.9 (5.9)	
Range	3–25	2–25	6–25	
Left testis				0.007 (5.00)
Mean (SD)	14.5 (6.7)	15.9 (5.9)	17.5 (6.0)	
Range	3–25	2–25	6–25	
Varicocele [No. (%)]	32 (45.1)	347 (53.3)	58 (55.2)	0.41 (χ^2^, 2.01)
Hypercholesterolemia [No. (%)]	16 (55.2)	117 (56.0)	12 (70.6)	0.52 (χ^2^, 1.41)
Hypertrigliceridemia [No. (%)]	7 (23.3)	26 (15.3)	2 (11.8)	0.52 (χ^2^, 1.47)
Impaired HDL-C [No. (%)]	20 (55.6)	80 (39.4)	11 (27.5)	0.04 (χ^2^, 6.26)
Alcohol [No. (%)]				0.93 (χ^2^, 0.85)
Abstainers	17 (23.9)	144 (22.2)	21 (20.0)	
Moderate drinkers	41 (57.8)	385 (59.1)	61 (58.1)	
Heavy drinkers	13 (18.3)	122 (18.7)	23 (21.9)	
Cigarette smoking [No. (%)]				0.53 (χ^2^, 1.25)
Current/ex smokers	25 (35.2)	208 (31.9)	29 (27.6)	
Never smokers	46 (64.8)	443 (68.1)	76 (72.4)	

BMI = body mass index; CCI = Charlson Comorbidity Index; LBW = low birth weight; NBW = normal birth weight; HBW = high birth weight;

*P value according to chi-square test or analysis of variance (ANOVA), as indicated

Conversely, patients with LBW shared a heavier burden of comorbidities (p = 0.003) and had a lower mean testicular volume (p = 0.007) compared to patients in the other groups, although there was no different prevalence of varicocele among the three patient groups divided according to BW. Moreover, LBW group showed a higher prevalence of impaired HDL-C values (p = 0.04) as compared with other groups; in contrast, no differences were observed in terms of impaired values of cholesterol or triglycerides among the groups.

Table 3 reports the characteristics and descriptive statistics of the hormonal milieu and seminal parameters of the cohort of patients according to BW categories. Regarding hormonal milieu, FSH values and tT levels varied according to BW; more specifically, LBW individuals had higher FSH values than both NBW (p = 0.02) and HBW patients (p = 0.005); conversely, normal and HBW group did not differ. Furthermore, fT levels were lower in the LBW group compared to the NBW (p = 0.003) and HBW (p = 0.04) groups, respectively; the two latter groups showed no significant differences. No significant differences were found regarding LH, InhB, AMH, E_2_, SHBG, PRL, and TSH values among groups (Table 3). Overall, sperm motility (p = 0.002) and normal morphology (p = 0.004) were significantly lower in the LBW group. To this regard, LBW individuals had lower sperm motility than both the NBW group (p = 0.003) and the HBW group (p = 0.043) while the normal and HBW group did not differ. Furthermore, sperm morphology was significantly lower in the LBW group compared to the NBW (p = 0.035) and HBW (p = 0.027) groups, while the latter two groups showed no difference. Overall, LBW men showed higher rates of asthenozoospermia (p = 0.02) and teratozoospermia (p = 0.03). Conversely, no differences were found in terms of semen volume and sperm concentration among groups.

**Table 3:** Characteristics and descriptive statistics of infertile patients’ hormonal milieu and seminal parameters according to birth weight.

	LBW	NBW	HBW	p value (F)*
FSH (mUI/mL)				0.02 (3.95)
Mean (SD)	12.5 (10.3)	8.9 (12.5)	7.0 (6.0)	
Range	0.1–57.0	0.6–19.8	0.3–29.9	
LH (mUI/mL)				0.82 (0.17)
Mean (SD)	5.9 (4.6)	5.6 (4.4)	4.3 (2.0)	
Range	1.70–26.0	0.10–34.0	0.1–9.6	
InhB (pg/mL)				0.18 (1.70)
Mean (SD)	88.0 (75.3)	116.1 (83.2)	107.3 (78.2)	
Range	0.7–244.7	0.01–439.4	0.5–291.0	
AMH (ng/mL)				0.93 (0.06)
Mean (SD)	5.96 (5.8)	6.4 (7.3)	6.1 (4.1)	
Range	0.2–21.0	0.1–96.4	0.4–17.5	
tT (ng/mL)				0.02 (3.75)
Mean (SD)	4.2 (1.8)	4.8 (1.8)	4.8 (1.7)	
Range	0.9–10.4	0.02–16.8	0.45–9.9	
E_2_ (pg/mL)				0.84 (0.17)
Mean (SD)	32.7 (18.8)	33.0 (13.6)	32.0 (13.8)	
Range	5.0–94.2	5.0–129.0	9.0–84.0	
SHBG (nmol/L)				0.99 (0.04)
Mean (SD)	32.3 (12.7)	33.1 (13.3)	32.3 (13.9)	
Range	15.0–60.0	6.0–95.0	12.0–75.0	
PRL (ng/mL)				0.86 (0.14)
Mean (SD)	9.5 (5.0)	9.8 (7.8)	9.3 (4.6)	
Range	2.80–23.8	1.01–135.0	2.6–23.9	
TSH (μUI/mL)				0.79 (0.23)
Mean (SD)	1.9 (1.1)	2.1 (3.8)	1.8 (0.9)	
Range	0.7–6.0	0.0–77.8	0.4–4.7	
Semen volume (mL)				0.21 (1.52)
Mean (SD)	3.0 (1.7)	3.1 (1.7)	2.8 (1.4)	
Range	0.4–10.5	0.2–12.0	0.3–7.3	
Semen volume <1.5 mL [No. (%)]	8 (16.0)	68 (12.1)	11 (12.2)	0.68 (χ^2^, 0.74)
Sperm concentration				0.82 (0.19)
Mean (SD)	31.4 (44.1)	33.9 (40.0)	31.9 (41.2)	
Range	0.5–190.4	0.5–266.0	0.5–226.0	
Sperm concentration ≤15x10^6^/mL [No. (%)]	30 (42.3)	257 (39.5)	42 (40.0)	0.91 (χ^2^, 0.28)
Progressive motility				0.002 (6.48)
Mean (SD)	20.3 (17.7)	27.5 (20.5)	26.4 (17.1)	
Range	0.0–60	0.0–94.0	0–65	
Progressive motility ≤32% [No. (%)]	52 (73.2)	369 (56.7)	55 (71.4)	0.02 (χ^2^, 8.32)
Normal morphology				0.04 (3.12)
Mean (SD)	2.62 (4.5)	5.98 (11.7)	6.82 (12.6)	
Range	0.00–85.0	0.00–80.0	0–45	
Normal morphology ≤4% [No. (%)]	54 (76.1)	422 (64.8)	59 (56.2)	0.03 (χ^2^, 6.75)

LBW = low birth weight; NBW = normal birth weight; HBW = high birth weight

*P value according to chi-square test or analysis of variance (ANOVA), as indicated

Tables 4 and 5 detail univariable and multivariable logistic regression models testing the associations between clinical predictors and either pathologic sperm parameters or the presence of health significant comorbidities (CCI≥1). At UVA, only higher FSH (p<0.001) and cigarette smoking (p = 0.04) were associated with pathologic sperm concentrations. Conversely, age, LBW, +varicocele, CCI≥1, BMI and alcohol consumption were not. Similarly, LBW was univariably associated with pathologic progressive motility (*p*≤0.02); both LBW (p<0.005) and FSH values (p = 0.003) were also significantly associated with pathologic sperm morphology. At logistic MVA, only FSH levels achieved independent predictor status for pathologic sperm concentration (p<0.001). Lower BW (p<0.04) achieved independent predictor status for lower sperm motility; both LBW and FSH values were independent predictors of pathologic sperm morphology (all p≤0.04). Only LBW significantly predicted higher CCI values (p<0.001).

**Table 4:** Univariable logistic regression models predicting pathologic sperm parameters according to WHO 2010 criteria and presence of comorbidities (CCI≥1) in the whole cohort of patients (n = 827).

	Oligospermia^a^	Low motility^b^	Pathologic morphology^c^	Comorbidity^d^
	OR (95% CI)	OR (95% CI)	OR (95% CI)	OR (95% CI)
	p	p	p	p
Age (years)	0.99 (0.97–1.02)	1.01 (0.97–1.04)	0.98 (0.96–1.01)	1.02 (0.91–1.02)
	0.72	0.82	0.43	0.27
LBW	1.52 (0.82–2.83)	2.87 (1.21–6.87)	2.71 (1.34–5.51)	3.62 (1.72–7.22)
	0.18	0.02	0.005	0.001
+Varicocele	1.03 (0.72–1.41)	1.41 (0.96–2.01)	1.01 (0.75–1.40)	0.81 (0.51–1.31)
	0.97	0.07	0.73	0.43
CCI≥1	1.82 (0.98–3.53)	1.12 (0.53–2.32)	1.31 (0.60–2.51)	—
	0.64	0.75	0.42	—
FSH	1.23 (1.16–1.31)	1.03 (0.99–1.07)	1.06 (1.02–1.13)	1.01 (0.99–1.01)
	<0.001	0.14	0.003	0.11
BMI	1.01 (0.97–1.13)	1.01 (0.95–1.07)	0.99 (0.94–1.01)	0.96 (0.89–1.04)
	0.45	0.59	0.755	0.34
Alcohol yes vs no	1.23 (0.75–2.06)	1.32 (0.77–2.24)	1.07 (0.65–1.78)	0.52 (0.27–0.96)
	0.42	0.30	0.78	0.06
Smoke yes vs no	1.46 (1.02–2.15)	0.96 (0.63–1.46)	1.48 (0.97–2.02)	0.94 (0.53–1.64)
	0.04	0.86	0.06	0.83

LBW = low birth weight; CCI = Charlson Comorbidity Index; BMI = body mass index;

^a.^ Sperm concentration <15x10^6^/mL

^b.^ Progressive motility <32%

^c.^ Normal morphology <4%

^d.^ Charlson Comorbidity Index ≥1

**Table 5:** Multivariable logistic regression models predicting pathologic sperm parameters according to WHO 2010 criteria and presence of comorbidities in the whole cohort of patients (n = 827).

	Oligospermia^a^	Low motility^b^	Pathologic morphology^c^	Comorbidity^d^
	OR (95% CI)	OR (95% CI)	OR (95% CI)	OR (95% CI)
	p	p	p	p
Age (years)	0.99 (0.95–1.03)	1.01 (0.97–1.05)	0.98 (0.95–1.02)	1.03 (0.97–1.03)
	0.68	0.61	0.55	0.15
LBW	0.44 (0.17–1.12)	3.17 (1.14–8.87)	2.06 (0.92–4.62)	3.71 (1.73–7.94)
	0.08	0.02	0.04	0.001
+Varicocele	1.13 (0.71–1.82)	1.66 (1.01–2.70)	1.03 (0.61–1.64)	0.70 (0.45–1.35)
	0.59	0.04	0.86	0.22
CCI≥1	1.54 (0.61–3.90)	0.70 (0.28–1.70)	1.28 (0.54–3.01)	--
	0.35	0.43	0.56	--
FSH	1.25 (1.17–1.34)	1.03 (0.97–1.06)	1.04 (1.05–1.09)	1.01 (0.91–1.12)
	<0.001	0.30	0.02	0.13
BMI	1.05 (0.98–1.13)	1.04 (0.97–1.12)	0.98 (0.95–1.05)	0.95 (0.82–1.01)
	0.16	0.22	0.66	0.33
Alcohol yes vs no	1.38 (0.65–2.93)	1.49 (0.72–3.10)	1.14 (0.57–2.29)	0.55 (0.21–1.22)
	0.40	0.27	0.70	0.14
Smoke yes vs no	1.26 (0.76–2.07)	0.86 (0.51–1.45)	1.21 (0.76–1.93)	0.75 (0.42–1.53)
	0.36	0.59	0.41	0.42

LBW = low birth weight; CCI = Charlson Comorbidity Index; BMI = body mass index;

^a.^ Sperm concentration <15x10^6^/mL

^b.^ Progressive motility <32%

^c.^ Normal morphology <4%

^d.^ Charlson Comorbidity Index ≥1

## Discussion

We assessed the prevalence along with the clinical and seminal impact of different categories of BW in a relatively large cohort of white-European men seeking medical attention for the first time for primary couple’s infertility. When compared with fertile men, infertile individuals showed a lower mean BW and a higher proportion of LBW. Moreover, infertile men had a higher mean BMI value and reported a greater proportion of clinically significant comorbidities (namely CCI≥1), as compared to fertile individuals. More specifically, we found that infertile men born with LBW had reduced sperm motility and reduced normal sperm morphology compared to NBW and HBW men. Individuals with LBW also showed lower tT levels but higher FSH values, compared with those in the other groups. Moreover LBW patients reported a higher rate of health significant comorbidities and reduced left testicular volume. As a whole, the current findings provide new evidence in support of the hypothesis that BW has an impact on gonadal function in postnatal life.

Our interest was fuelled by existing controversies regarding the relationship between the developmental environment and postnatal reproductive function in men [8–15]. In this context, we found that the BW of infertile men was significantly lower than that of fertile individuals. Our findings support previous evidence indicating that LBW is associated with reduced men’s gonadal function in the elderly. Focusing on the endocrine part of the testis function, Cicognani *et al*. for instance reported that SGA individuals have a pituitary-gonadal axis that tends toward hypogonadism [8]. A positive association with BW and sex steroids in young adulthood has also been reported [10]. Moreover, various studies have shown increased serum FSH levels in SGA boys [8,9]. In contrast, other authors have not found significant relationships between BW and gonadal function [12,15]. Overall, our findings show that in a homogeneous cohort of primary infertile patients impaired sex steroids values, in terms of circulating tT and FSH levels, were more frequently reported in LBW men as compared with those in the NBW and HBW groups. These results strongly support the fetal programming hypothesis declaring that some cardiovascular, metabolic and endocrine set points could be alterated by fetal adaptation to adverse intrauterine environment, which could be expressed by the LBW individuals [29,30].

Few studies have investigated the impact of BW on seminal parameters of fertile and subfertile individuals. Francois *et al*. showed that subfertile men had a LBW while men with normal semen analysis reported a NBW [7]. However, that study had a major limitation because BW details were obtained using questionnaires, thus leading to potential recall bias. More recently Faure *et al*. found that BW was associated with sperm DNA fragmentation and inversely correlated with total sperm count [11], highlighting the importance of the in utero environment for the development of male reproductive function. On the contrary, other authors have failed to find an association between BW and semen quality in adult life [14–16]. In our cohort of infertile men, those with LBW showed reduced sperm motility and reduced normal sperm morphology compared to NBW and HBW men. Conversely no differences were observed in terms of sperm concentration or sperm volume between groups.

To the best of our knowledge, this is the first naturalistic, cross-sectional, observational study in which a relatively large cohort of infertile individuals underwent a comprehensive clinical, endocrine and seminal evaluation. Of importance, we found a statistically significant association between BW and impaired seminal parameters. Although BW represents only a proxy of the intrauterine factors acting on the development of the gonadal system, we speculate that it may have an influence on semen quality in adulthood.

From a clinical standpoint, higher BW is generally an indication of a more favourable intrauterine environment, although some exceptions have been identified, thus including the macrosomia exhibited by infants of diabetic women [31]. On the contrary, lower BW occurs due to inadequate intrauterine conditions that lead to abnormal foetal development. Strong evidence exists from several studies indicating that individuals born with a LBW are more likely to present cardiometabolic complications in later life [6]. Furthermore, LBW has been associated with lower lean body mass and greater central obesity [31], although the association between lower BW and later BMI remains at least controversial [32]. In our cohort of individuals, infertile men had higher BMI values and included a greater proportion of BMI suggestive of obesity compared with fertile men. Palatilanou *et al*. reported, instead, that individuals born with HBW had an increased risk of being obese in later life, being affected by diseases of the heart and circulation, but were not at an increased risk for the development of type 2 diabetes mellitus [33]. Our finding that HBW men more frequently reported a BMI value suggestive for NIH class ≥1 obesity is thus in accord with previous reports. Interestingly, LBW men more frequently showed even impaired HDL-C values, suggesting implications of restrictive in utero condition on the metabolic system in adulthood.

Of clinical relevance, men with LBW reported a higher prevalence of health significant comorbidities, depicted by the CCI, as compared with the other groups. Despite the known association between the weight at birth and cardiometabolic diseases [6], no one has previously investigated the link between general health status and LBW in primary infertile patients. It is already known that MFI men have an increased risk of developing testicular germ cell tumor, colorectal cancer, melanoma, and prostate cancer [34–36]. More precisely, Eisenberg *et al*. showed that men with azoospermia have an increased risk of subsequently developing cancer, suggesting a possible common aetiology between azoospermia and cancer development [36]. Moreover, MFI has been associated with a higher CCI, which may be considered a reliable proxy of lower general health status, regardless of the etiology of pure male infertility [37]. Recently, Ventimiglia *et al*. found a strong association between a decreased general health status (coded by the CCI) and impaired gonadal function in infertile men [38]. Likewise, Eisenberg *et al*. reported a relationship between medical comorbidities and male semen production [39]. As we found that LBW is an independent predictor of poorer health status (namely CCI≥1) we can speculate that LBW could represent a risk factor for the malfunctioning of the male reproductive system as a whole.

One major limitation of this study is related to the fact current that data come from a hospital-based centre assessing primary infertile men only, thus highlighting the importance of an external validation of the current results. Second, although the study provides original and novel findings, our relatively small cohort of white-European men could limit the meaningfulness of the findings themselves. In this context, though the rigorous homogeneity of the sample in racial terms may represent strength, to ensure the generalizability of the results, a larger population-based survey should be employed to validate the current findings. Third, we have no data on the seminal characteristics of the fertile population, so investigating the impact of BW in the healthy individuals was not possible. Finally, although all enrolled patients reported being born at term their exact gestational age was actually not available in the birth records to confirm these reports.

## Conclusion

These cross-sectional analyses in a relatively large homogeneous white-European cohort of primary infertile men (restricted to non-interracial infertile couples) showed novel evidence of a higher prevalence of LBW as compared with a comparable cohort of fertile individuals. Moreover, LBW not only is associated with qualitative alterations of the semen, but it also associated with a decreased overall health status.

## Supporting Information

S1 Dataset Dataset containing data for statistical analyses. (XLS)
